# Phylogeography and Symbiotic Effectiveness of Rhizobia Nodulating Chickpea (*Cicer arietinum* L.) in Ethiopia

**DOI:** 10.1007/s00248-020-01620-8

**Published:** 2020-10-24

**Authors:** A. H. Gunnabo, J. van Heerwaarden, R. Geurts, E. Wolde-meskel, T. Degefu, K. E. Giller

**Affiliations:** 1 Plant Production Systems Group, Wageningen University and Research, Wageningen, The Netherlands; 2 Laboratory of Molecular Biology, Department of Plant Science, Wageningen University, Wageningen, The Netherlands; 3 World Agroforestry Centre (ICRAF), Addis Ababa, Ethiopia; 4 International Crops Research Institute for the Semi-Arid Tropics, Addis Ababa, Ethiopia

**Keywords:** Genetic diversity, Genospecies, Haplotypes, Mesorhizobial strains, Nucleotides, Spatial patterns

## Abstract

Chickpea (*Cicer arietinum *L.) used to be considered a restrictive host that nodulated and fixed nitrogen only with *Mesorhizobium ciceri* and *M. mediterraneum*. Recent analysis revealed that chickpea can also establish effective symbioses with strains of several other *Mesorhizobium* species such as *M. loti*, *M. haukuii*, *M. amorphae*, *M. muleiense*, etc. These strains vary in their nitrogen fixation potential inviting further exploration. We characterized newly collected mesorhizobial strains isolated from various locations in Ethiopia to evaluate genetic diversity, biogeographic structure and symbiotic effectiveness. Symbiotic effectiveness was evaluated in Leonard Jars using a locally released chickpea cultivar “Nattoli”. Most of the new isolates belonged to a clade related to *M. plurifarium*, with very few sequence differences, while the total collection of strains contained three additional mesorhizobial genospecies associated with *M. ciceri*, *M. abyssinicae* and an unidentified *Mesorhizobium* species isolated from a wild host in Eritrea. The four genospecies identified represented a subset of the eight major *Mesorhizobium* clades recently reported for Ethiopia based on metagenomic data. All Ethiopian strains had nearly identical symbiotic genes that grouped them in a single cluster with *M. ciceri*, *M. mediterraneum* and *M. muleiense*, but not with *M. plurifarium*. Some phylogeographic structure was observed, with elevation and geography explaining some of the genetic differences among strains, but the relation between genetic identity and symbiotic effectiveness was observed to be weak.

**Electronic supplementary material:**

The online version of this article (10.1007/s00248-020-01620-8) contains supplementary material, which is available to authorized users.

## Introduction

Chickpea (*Cicer arietinum* L.) is one of the most important grain legumes globally, ﻿with a total production of 11.6 million tons from an area of 13.2 million ha and with a productivity of 0.88 tons ha^−1^ [1]. Ethiopia is considered a secondary centre of diversity for chickpea [2] with cultivation dating back to 500 BCE [3, 4]. The country currently cultivates an average of 0.23 million ha (data for 2017), with a total estimated production of 0.43 million tons, making Ethiopia the fourth major producer in the world after India, Australia and Turkey. In Ethiopia, the crop is mainly grown on vertisols on progressively declining residual soil moisture towards the end of rainy season [5]. Here, average yields of 1.84 tons per ha [6] are achieved, far below﻿ the potential yield of 5.0 tons per ha [5]. The relatively poor grain yield is attributed to poor soil fertility combined with a lack of fertilizers [2], related to the common notion among farmers that legume crops do not need nutrient inputs [7]. The latter idea may derive in part from the fact that grain legumes like chickpea are able to fix atmospheric nitrogen through symbiosis with rhizobia. This presumes that nitrogen will not be limiting as long as compatible bacteria are present in the soil. Still, there is ample evidence that chickpea yields can be enhanced by inoculation with elite rhizobia [8]. The success of inoculation depends on both the biological properties of the inoculant strain and on the composition of native rhizobial populations in the target soils. It is therefore relevant to understand the taxonomy and diversity of chickpea rhizobia native to Ethiopia.

Chickpea is a restrictive host, nodulated by a single genus of rhizobia, *Mesorhizobium*, that primarily includes *M. ciceri, M. mediterraneum* [9, 10], *M. muleiense* [11] and *M. wenxiniae* [12]*.* Several other strains such as *M. loti*, *M. haukuii*, *M. amorphae* and *M. plurifarium* [13], which are the natural symbionts of other legumes, can also effectively nodulate chickpea. These strains might have obtained symbiotic genes from *M. ciceri* and *M. mediterraneum* through horizontal gene transfer (HGT), since they share identical symbiotic genes [14–16]. ﻿These symbiotic genes are clustered on chromosomal islands in the genome of *Mesorhizobium* [17, 18], while they are found on transmissible plasmids in *Rhizobium* and *Sinorhizobium* [17, 19]. Bacterial genomes are in a constant state of flux, and any DNA segment may have the opportunity of HGT in bacteria population [20]. The HGT can be triggered by environmental stress conditions [21]; occur either by transduction, transformation or conjugation [20]; and could involve homologous recombination that results in the exchange of orthologous genes between evolutionary lineages and the transfer of a gene from one evolutionary lineage to another [22]﻿. There is evidence of the HGT in *S. medicae* and *S. meliloti* [22] and in other rhizobia species including the mesorhizobia as reviewed by Andrews et al. [21]. The HGT among rhizobia can influence the genetic diversity and distribution, and this could be phenomenal in Ethiopia, where there exists large genetic diversity of hosts and potential exchange of the host plant seeds that carry the rhizobia [3].

Relatively little is known of the taxonomic composition and patterns of diversity of native *Mesorhizobium* in Ethiopia. At the global scale, the genetic distance among *Mesorhizobium* strains was shown to correlate with geographic distance [23], contrary to the Baas-Becking hypothesis that states that “everything is everywhere, the environment selects” [24]. This could indicate that diversity is limited at the country level compared with global populations. On the other hand, it is conceivable that regions, such as Ethiopia with high chickpea diversity and long cultivation history, harbour genetically diverse rhizobia [25, 26]. Also, local contrasts in environmental factors such as pH, temperature, moisture, salinity, elevation and the presence of host plants are known to influence the distribution of the rhizobia [27–29] and may determine patterns of genetic diversity in Ethiopian *Mesorhizobium* species that can nodulate chickpea [23]. A recent, comprehensive study on worldwide *Mesorhizobium* diversity revealed that the strains compatible with Ethiopian chickpea represent a relatively wide range of clades, both at the whole-genome level and at the level of symbiotic genes [23]. This result apparently contradicts an earlier small-scale study that found only few clades, particularly for the symbiotic genes among 18 strains [30]. One explanation for the discrepancy lies in the fact that many of the strains in the former study were identified using direct sequencing on nodules rather than on cultivated strains as were used in the latter study. It is known that culture-based studies may underestimate the diversity present in bacterial communities compared to culture-independent metagenomic approaches that use DNA extracted directly from soil or nodules [31, 32]. This would imply that only a subset of nodulating bacteria are suitable for cultivation and eventual inoculant production, suggesting that a lot functional diversity would necessarily remain untapped. On the other hand, the observed lack of diversity among cultivated strains may simply reflect a sample of insufficient size or geographic scope in the smaller study, in which case additional sampling would be expected to yield additional diversity.

Here, we report on the taxonomy and patterns of genetic diversity in a set of 21 additional *Mesorhizobium* strains sampled from Ethiopia, which we combine with published data on 18 strains [30]. We thereby aim to evaluate the genetic diversity of chickpea-nodulating mesorhizobial strains isolated from Ethiopian soils and to assess if spatial and environmental patterns among these strains exist that may contribute to taxonomic differences between geographically restricted samples. Finally, we aim to determine whether relationships exist between the taxonomy or geography of rhizobia and their symbiotic effectiveness, which is of great relevance for future bioprospecting efforts.

## Materials and Methods

### *Mesorhizobium* Strains Used for Molecular Characterization

**Fig. 1:**
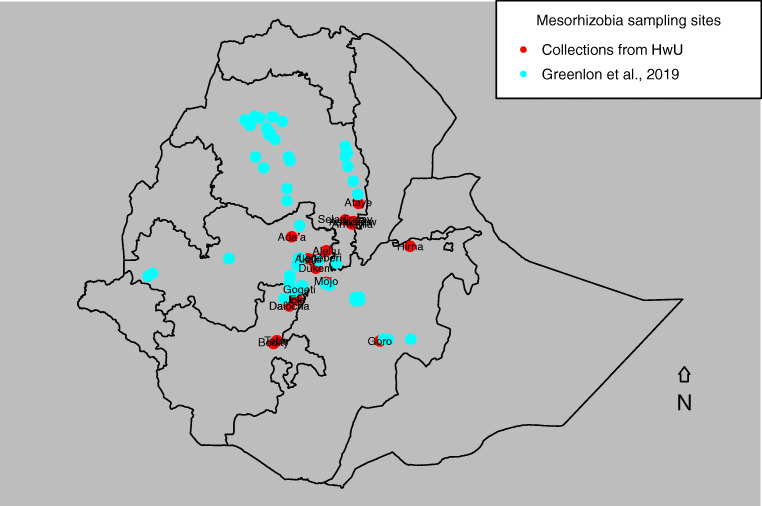
Sampling sites of *Mesorhizobium* strains collected from Ethiopia by Greenlon et al., 2019 and HwU 3 (Hawassa University, Soil Microbiology Laboratory) and included in the current analysis

Chickpea-nodulating mesorhizobial strains, previously isolated from soils sampled from different chickpea-growing areas in Ethiopia, were characterized by molecular methods using housekeeping genes (21 strains) and symbiotic genes *nodC* and *nifH* (32 strains) (Table S1). The areas in Central and Southern Ethiopia, from where the soil samples were collected and used to trap these strains [33, 34], have never been inoculated with commercial strains [30, 35]. Figure 1 shows the sites from where the current and Greenlon et al. [23] strains were trapped. In the molecular analysis, some of the strains that were positive for *nodC* and *nifH* were not amplified by housekeeping genes, and inversely strains that had amplicons for housekeeping genes were not amplified by the symbiotic genes. These strains were collected by plant trap method in screening house for the purpose of exploring genetic and symbiotic diversity. We reconstructed the phylogeny of the newly characterized strains together with 18 published sequences of *Mesorhizobium* strains from the country [30] and 25 reference strains previously used to relate mesorhizobia collection from Ethiopia [23]. The presence of spatial patterns among them over biogeography and their genetic affiliation with symbiotic effectiveness were evaluated. These strains were retrieved from collections maintained at the Laboratory of Soil Microbiology, Hawassa University. The strains were checked for viability and purity by growing on yeast extract mannitol agar (YMA) medium containing Congo Red (CR) dye [33, 34]. Contaminated strains were purified and renamed by adding lower case letters after the original codes, while none-viable ones were discarded. All the test strains including references were prepared in 1.5 ml Eppendorf tubes containing 300 μl of 15% glycerol and 700 μl of broth culture and maintained in a refrigerator adjusted to − 20 °C at the Hawassa University.

### Molecular Characterization of the Rhizobial Isolates

We amplified partial 16S rRNA and *recA* housekeeping genes and *nifH* and *nodC* partial symbiotic genes directly from colony suspensions following procedures we described previously [36]. We additionally amplified a partial gene of *atpD* using primers atpDf (273–294 target gene position): 5′- SCT GGG SCG YAT CMT GAA CGT-3′ and atpDr (748–771 target gene position): 5′- GCC GAC ACT TCC GAA CCN GCC TG-3′ with the same PCR conditions used for *gyrB* and *rpoB* genes [36]. For all the PCR reactions, PCR master mix was prepared by thoroughly mixing 17.4 μl MQ water, 2.5 μl (10x) Dream Taq buffer, 1 μl (10 mM each forward and reverse primers) and 0.1 μl (5 U/μl) Dream Taq DNA polymerase enzyme (Thermo Fischer Scientific Inc.) to make 23 μl reaction volume [36]. The final reaction volume was made 25 μl by adding 2 μl of the rhizobial colony suspension and amplified using a PCR (BioRad Company). The PCR products were cleaned using Thermo-Scientific PCR product cleaning kit and sequenced by Macrogen Inc. (the Netherlands). *GlnII* and *rpoB* genes were excluded from this analysis due to failure to amplify some of the strains using primers targeting *glnII* and *rpoB*. In addition to this, different regions of *nifH* were sequenced for both current and previous strains [30], while *nodA* gene was amplified instead of *nodC* in the previous analysis. Thus, only currently characterized and reference strains were analysed using *nodC* and *nifH* genes. The accession numbers of the sequences are as follows: MT381518 - MT381538 for 16S rRNA, MT381539 - MT381559 for *atpD*, MT381560 - MT381593 for *gyrB*, MT381594 - MT381614 for *recA*, MT381615 - MT381645 for *nodC* and MT381646 - MT381678 for *nifH*.

### Phylogenetic Analysis

The quality of the DNA sequences was checked and edited by BioEdit Sequence Alignment Editor. The edited sequences were compared to GenBank database using the online nucleotide BLAST method (https://blast.ncbi.nlm.nih.gov/) to check if the right gene is sequenced and to which *Mesorhizobium* species it belongs. Multiple nucleotide sequence alignments were carried out using the CLUSTAL W program as we described previously [36] and concatenated in R 3.6.1 [37]. Phylogenetic trees of each and concatenated housekeeping genes were reconstructed using Tamura and Nei [38] Model (TN93) with Gama distribution (+G) and invariants among sites (+I) under maximum likelihood method in R using *ape* package. The robustness of the tree topology was calculated from bootstrap analysis with 500 replications of the sequences for maximum likelihood. The *nodC* phylogeny was reconstructed using Tamura 1992 (T92) [39] + G + I model and *nifH* was reconstructed using T92 + G model with 1000 bootstrap analysis under the maximum likelihood criterion. The percentage similarity of the genes was estimated using BioEdit software.

### Evaluation of Symbiotic Effectiveness

The symbiotic effectiveness of the strains was evaluated in modified Leonard Jars (LJ) using a chickpea variety “Nattoli”. The modified LJ preparation and RCBD experimental design with three replicates were used to evaluate the symbiotic effectiveness following the same procedures and growing conditions reported previously [36]. Nodule number, nodule dry weight, shoot dry weight and root dry weight were measured on all plants. These correlated traits were scaled, and the first principal component was used as a synthetic measure of overall symbiotic response to be used in subsequent analyses.

### Data Analysis

Genospecies were assigned to major genetic clusters identified in the comprehensive study by Greenlon et al. [23] based on sequence similarity to their published reference strains or, in one case, sequence similarity of our reference strains to their published sequences. Significance of differences in taxonomic composition between different data subsets and between studies was evaluated by a Chi-squared test. Significance of differences in genospecies diversity were evaluated by comparing the observed values for Shannon and Simpson’s diversity indices with those calculated for 10,000 random samples of 39 strain taken, with replacement from Greenlon et al.’s [23] 98 cultured strains from Central Ethiopia (Fig. 1).

Genetic diversity was calculated at nucleotide, haplotype (locus) and species levels of genomic hierarchy as follows: at the species level, Shannon and Simpson indices [40] were calculated based on genospecies using the *vegan* package in R 3.6.1. At the level of individual genes, allele sharing distance was calculated and averaged across loci, while at the nucleotide level, the pairwise proportion of different sites was calculated [41].

The presence of biogeographic structure among mesorhizobia was assessed using Mantel tests that correlate geographic and genetic distances of nucleotides, haplotypes and species [42] using *ape* package in R. We controlled the covariate effect of elevation on the biogeographic structure by using a partial Mantel test [42]. Additionally, we performed regression analysis (using R’s *lm* function) to test the relation between genetic variation, as captured by individual orthogonal coordinates obtained from principal coordinates analysis (PCoA) of the genetic distance matrix [43], to altitude and geographic position. With respect to the latter, the first two principal coordinates of the geographic distance matrix were used rather than latitude and longitude, to account for the fact that many isolates were sampled along a linear south-west to north-east transect. Significance was tested with an F-test using the *anova* function in R.

To describe the dependence of genetic diversity as a function of distance, a sliding-window resampling analysis was performed. Windows of 40-km width were moved at 10-km intervals, assuming only few haplotypes dominate within this distance [44]. For each interval, diversity statistics were calculated for a randomly selected pair of strains within the specified distance interval. Genetic diversity values of strains sampled at random distances were calculated for comparison.

The relation between symbiotic effectiveness and genetics was tested by fitting a linear mixed model with strain and replicate as random terms, using the aforementioned genetic/geographic principal coordinates as explanatory variables and the first principal component of symbiotic traits as response variable.

## Results

### Phylogenetic Analysis

DNA sequence data was obtained for a total of 89 strains (21 newly cultured, 18 previously published, 25 reference strains from Greenlon et al. [23] and 25 additional type strains) for the following 16S rRNA (1040 bp), *atpD* (400 bp) and *recA* (319 bp) housekeeping genes. For the symbiotic genes *nodC* and *nifH*, 32 newly cultured strains were analysed. The three housekeeping genes were concatenated into a single alignment of 1742 bp positions. The sequence alignment statistics for concatenated and individual loci is given below in Table 1. Concatenated housekeeping (HK) genes revealed higher variable regions and parsimony information for the sequence alignments. Among the individual loci, *nodC* had the most parsimony informative sites but did not appear to differentiate the Ethiopian strains into separate clusters as in the case of housekeeping genes (see below). We used the HK alignment for genotypic diversity and biogeographical analysis of the strains and for comparison with symbiotic performance.

**Table 1:** Sequence statistics for each locus or concatenated sequence alignments

Locus	No. of sequences	Alignment length	Conserved region	Variable region	Parsimony information	Singletons	Nucleotide substitution selection model based on ML fits in MEGA7
16s rRNA	89	1045	921	122	53	69	K2 + G + I
*atpD*	89	390	255	132	104	28	T92 + G
*recA*	89	307	200	105	75	30	T92 + G
*nifH*	63	381	243	133	100	32	T92 + G
*nodC*	64	494	195	297	235	62	T92 + G + I
HK	89	1742	1376	359	232	127	TN93 + G + I

*HK* concatenated alignments of 16s rRNA, *atpD* and *recA*, *K2* Kimura 2-parameter model, *T92* Tamura (1992) model, *TN93* Tamura and Nei (1993) model, *G* gamma distribution, *I* invariant rate among sites

### Multilocus Sequence Analysis

**Fig. 2:**
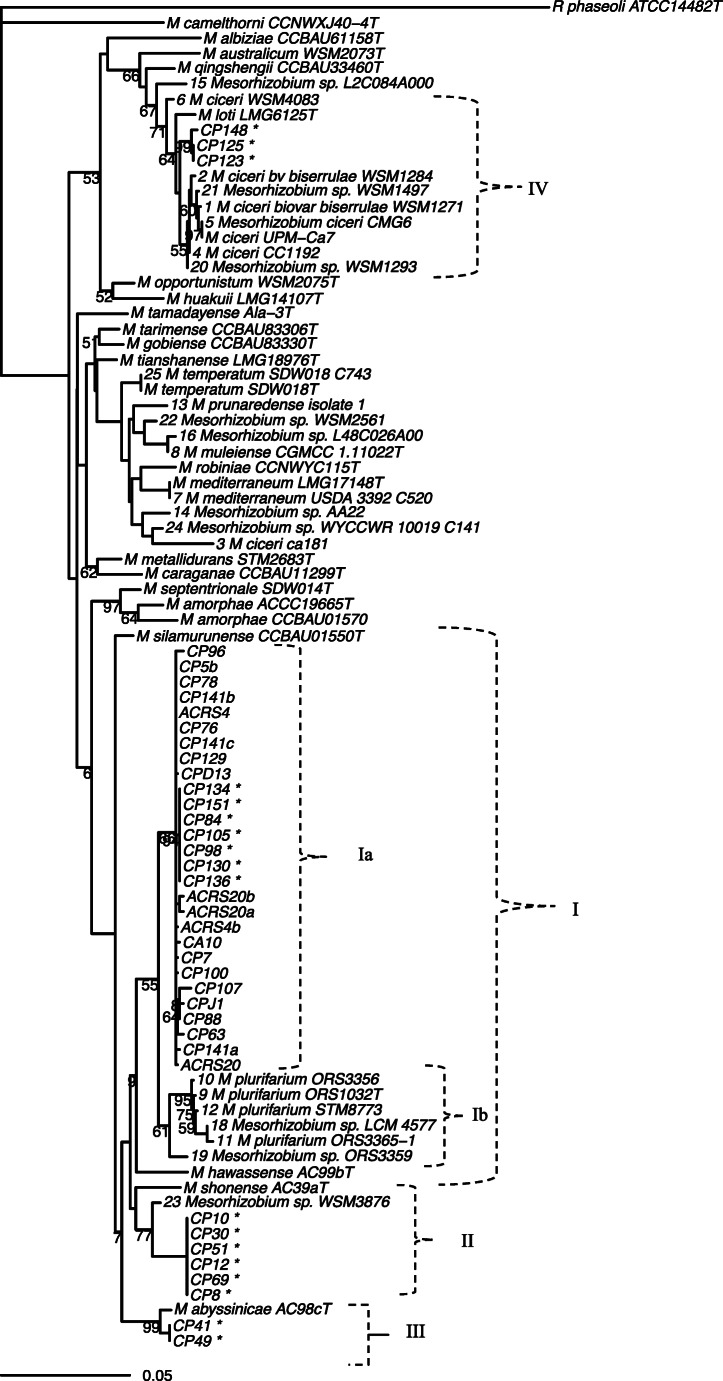
Phylogeny of multilocus sequence analysis (MLSA) of concatenated housekeeping genes of 16S rRNA, *atpD* and *recA* reconstructed using TN93 + I + G model in R. Strains with “*” were obtained from previous culture collection [30], and “T” at the end of some reference strains indicate type strains for that species

The concatenated housekeeping (HK) genes or multilocus sequence analysis (MLSA) clustered all of the currently and previously sequenced mesorhizobial isolates from Ethiopia into four distinct genospecies, recognized as genospecies I–IV (Fig. 2). Genospecies I contained all current and five of the previous strains. This genospecies formed a monophyletic cluster with *M. plurifarium* ORS1032^T^ with moderate bootstrap (BT) support (90%). At sequence similarity level, individual strains in this cluster had 97.1–99.7% average nucleotide identity (ANI) with ORS1032^T^ (genospecies Ib) and 95.3–97.1% ANI with *M. hawassense* AC99b^T^, which represents a basal clade in cluster I. The local Ethiopian strains in this cluster were segregated into subcluster Ia that was supported with 100% BT. The strain AC99b^T^ was previously isolated from Ethiopian soil using ﻿*Sesbania sesban* as a trap host [45], while ORS1032^T^ was isolated from an ﻿*Acacia senegal* nodule in Senegal [46]. The second cluster contained only previously characterized local Ethiopian strains and was assigned to a published but uncharacterised *Mesorhizobium* sp*.*, WSM3876, originally isolated in Eritrea from a shrub *Biserrula pelecinus* [47]. *Mesorhizobium* strains isolated from this host legume were previously reported as ﻿*Mesorhizobium ciceri* bv. *biserrulae*, and the strain WSM3876 was known to nodulate chickpea [47–49]. The local strains within this cluster shared 98.5–98.6% ANI with WSM3876. This clade was previously identified as being most closely related to *M. shonense* AC39a^T^ [30] that was isolated from Ethiopia [45]. It shares 97.6% ANI, but the node joining AC39a^T^ to the WSM3876 [23] clade had low bootstrap support (77%) using our sequence data. The third cluster, containing only two previously characterized local Ethiopian strains, was assigned to *M. abyssinicae* AC98c^T^, which was trapped from ﻿*Acacia abyssinicae* in Ethiopia [45]. The strains in the third genospecies cluster were supported with 100% BT value and shared 95.5% ANI with AC98c^T^. The last genospecies cluster contained three of the previously sequenced strains that were assigned to *M. loti* LMG6125^T^, *M. ciceri* UMP-Ca7^T^ and other related species with lower BT support (82%). The strains in this cluster shared 97.0–99.8% ANI with UMP-Ca7^T^ and similarly share 97.0–99.8% ANI with LMG6125^T^. The reference strains in the fourth genospecies cluster were previously known to effectively nodulate chickpea, while the reference strains in genospecies I, II and III were not known to effectively nodulate chickpea. However, Greenlon et al. [23] and Tena et al. [30] detected strains related to genospecies clusters I, II and III from root nodules of chickpea in Ethiopia, indicating that these strains are symbionts of chickpea. On the other hand, the close relatedness of the rhizobia nodulating *A. senegal* and strains of the first three genospecies that nodulated chickpea may reflect the genetic exchange between the tree legume and chickpea-nodulating mesorhizobial populations.

### Symbiotic (Sym) Gene Analysis

**Fig. 3:**
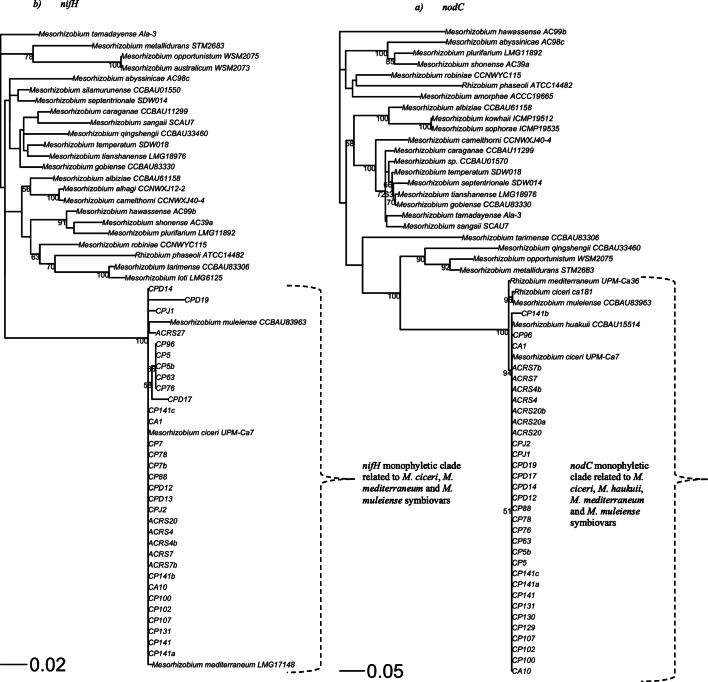
Symbiosis gene phylogenies reconstructed using *nifH* and *nodC* gene sequences

The separate analysis of the symbiotic genes *nodC* and *nifH* produced phylogenies that classified all of the Ethiopian strains into a single symbiovar, supported with 100% BT values (Fig. 3). In these phylogenies, the local strains were assigned with previously known chickpea-nodulating type strains such as *M. ciceri* UPM-Ca7^T^, *M. mediterraneum* UPM-Ca36^T^ and *M. muleiense* CCBAU 83963^T^ (Fig. 3a, b). In addition to these type strains, a reference strain *M. haukuii* CCBAU 15514 (isolated from ﻿*Astragalus sinicus* [50]) was also assigned with the same symbiovar in the *nodC* phylogeny (Fig. 3b); however, we found no *nifH* sequence for this strain in GenBank and excluded it from *nifH* phylogeny. The local strains that associated with the tree legume nodulating rhizobia (*M. plurifarium* ORS1032^T^, *Mesorhizobium sp.* WSM3876, *M. abyssinicae* AC98c^T^, *M. silamurunense* CCBAU01550^T^, *M. hawassense* AC99b^T^ and *M. shonense* AC39a^T^) in the MLSA phylogeny (Fig. 2) shared similar symbiotic genes with true symbionts of chickpea rhizobia (Fig. 3). This implies that the genetic transfer occurred from *Mesorhizobium* strains nodulating chickpea to *Mesorhizobium* strains that nodulate the tree legumes and enabled them to nodulate chickpea.

### Genetic Diversity of Chickpea Rhizobia

Genetic diversity of the chickpea-nodulating local Ethiopian strains was compared to that of other members of the *Mesorhizobium* genus (Table 2). In all cases (chickpea-nodulating and non-chickpea-nodulating *Mesorhizobium* strains), the reference strains were found to be genetically more diverse than the local Ethiopian strains at all levels of genetic hierarchy for both symbiotic and housekeeping genes. Among the individual genospecies, as defined based on housekeeping genes, strains that belong to *M. loti* LMG6125^T^ (genospecies IV) were genetically more diverse than any of the other genospecies at nucleotide and haplotype levels. Strains belonging to *Mesorhizobium* sp. WSM3876 revealed the least genetic variation compared to others. Analysis of symbiotic genes resulted in only a single symbiovar and genetic diversity within it to be very small (0.001). This shows that the symbiotic genes are relatively more conserved genes than the housekeeping genes, which in turn mean that only one symbiotic island has been shared among all of the mesorhizobia. The latter is in line with the findings of Garrido-Oter et al. [51] who identified a single gain of symbiosis genes in the genus *Mesorhizobium.* That is why, the symbiotic (accessary) genes are often used to determine host specificity, while the housekeeping (core) genes are used to infer phylogenetic relationship among rhizobial strains [52].

**Table 2:** Genetic diversity per source of strains

Sources of variation		Nucleotides	Haplotypes	Shannon	Simpson
HK	Reference vs local strains				
	^a^ References	0.0394	1.000	3.163	0.957
	^b^ Chickpea Ref	0.0308	1.000	1.906	0.834
	^c^ Non-chickpea Ref	0.0433	1.000	2.833	0.941
	^d^ Isolates	0.0158	0.920	0.613	0.335
	Greenlon	0.0307	1.000	1.116	0.602
	Total	0.0319	0.984	2.496	0.817
nodC	^a^ References	0.1828	0.9947	3.3322	0.9643
	^b^ Chickpea Ref	0.1438	0.9722	2.1972	0.8889
	^c^ Non-chickpea Ref	0.1677	0.9942	2.9444	0.9474
	^d^ Isolates	0.0013	0.1230	0.2771	0.1191
	Total	0.1342	0.7192	2.3201	0.7250
Species	Genospecies/symbiovars	MLSA		*nodC*	
		Nucleotide	Haplotype	Nucleotide	Haplotype
	*M. plurifarium* ORS1032^T^ (I)	0.00608	0.925	NA	NA
	*Mesorhizobium sp. WSM2876* (II)	0.00350	0.286	NA	NA
	*M. abyssinicae* AC98c^T^ (III)	0.00398	0.667	NA	NA
	*M. loti* LMG6125^T^ (IV)	0.00785	0.974	NA	NA
	*M. ciceri/M. mediterraneum*	NA	NA	0.001	0.069

^a^All the reference strains included in the analysis

^b^Reference strains that were known to nodulate chickpea

^c^Reference strains which were not able to nodulate chickpea

^d^Isolates are local strains or the test strains

A comparative diversity analysis at the level of major clades also revealed that the current sample had levels of taxonomic diversity far below what was observed for strains from Central Ethiopia isolated by Greenlon et al. [23]. The values of Shannon and Simpson’s diversity indices were 1.116 and 0.602 for Greenlon’s sample respectively versus 0.613 and 0.335 in the presents sample (Table 2), a difference that was highly significant in both cases (*p* < 0.0001).

### Biogeography of Chickpea-Nodulating Mesorhizobia in Central Ethiopia

**Fig. 4:**
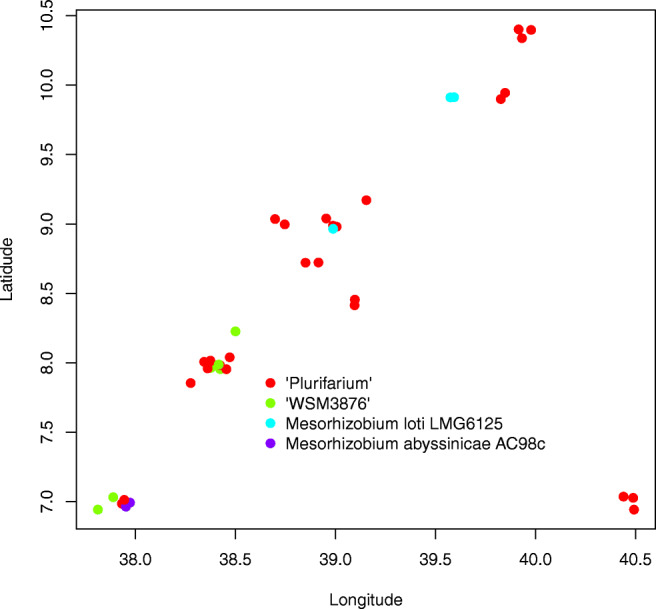
Spatial distribution of Mesorhizobia over space. Colour difference in the scatter plot indicates relative distribution of mesorhizobial genospecies to each other in space. “Plurifarium” represents *R. plurifarium* genospecies (see cluster I in Fig. 1), and “WSM3876” represents *Mesorhizobium* WSM3876 (see cluster II in Fig. 1).

The existence of phylogeographic structure in *Mesorhizobium* is of relevance for guiding future bioprospecting efforts in Ethiopia. Figure 4 shows the spatial distribution of the four identified genospecies. It is clear that the *M. plurifarium* genospecies is geographically widespread, occurring throughout the sampling region. By contrast, the two *M. abyssinicae* strains were found in a single location and the *Mesorhizobium* sp. WSM3876 genospecies in three locations in the south-western part of the sampling range. The three strains representing the *M. loti* genospecies were obtained from two locations in the highlands in the central and north-eastern part of the range. Notwithstanding the dominance of the *M. plurifarium* genospecies, four out of the sixteen locations contained more than one genospecies.

**Fig. 5:**
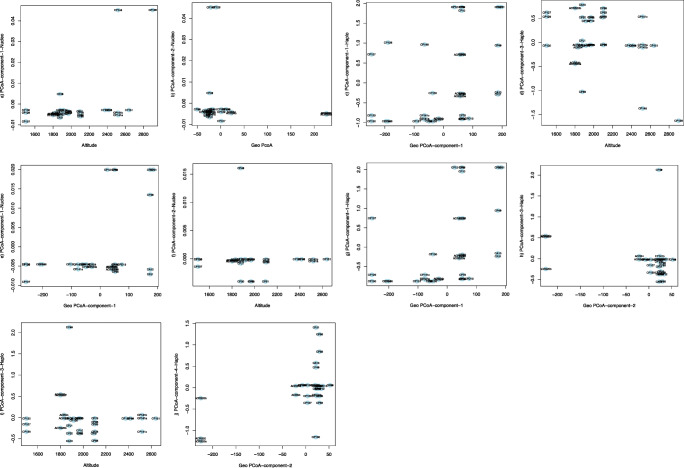
Effects of geographic and altitude components on the distribution of strains at nucleotide and haplotype levels of genetic level. On the *x*-axis, geographic or altitudinal distance components by which strain distribution is significantly affected. On the *y*-axis, principal coordinate distances at nucleotides or haplotypes that were influenced by the geographic distances is presented. Strains were scattered within the coordinate plane, showing their dispersal in space

Formal testing of phylogeographic structure supported the observation that the spatial distribution of rhizobia was non-random. The (partial) Mantel tests revealed significant correlations between altitude and nucleotide distance and geographic distance and haplotype dissimilarity (Table 3). PCoA regression analysis (Table S2) confirmed the relation between altitude and genetic distance and revealed that this relation was driven by the three strains assigned to the *M. loti* genospecies that were isolated from two sites above 2400 m.a.s.l (Fig. 5a,b). Removing these strains indeed eliminated the significant relation with altitude (Table S3, Fig. 5e–j). Both at the haplotype and nucleotide level, there were significant associations between genetic principal coordinates and geographic principal coordinates (Table S2, Fig. 5a–d). This was particularly evident upon removal of the *M. loti.* For this reduced set, the first coordinate derived from the nucleotide distance matrix associated strongly with the first geographic coordinate due to strains of the *Mesorhizobium* sp. WSM3876 and *M. abyssinicae* genospecies, which were restricted to the extreme south-west of the range as noted above. A similar pattern was observed for the components derived from the haplotype sharing dissimilarity matrix, but, in this case, an additional significant association between the third genetic and the second geographic coordinate was driven by three *M. plurifarium* strains ACRS20, ACRS20a and ACRS20b which were found in a single location and show few nucleotide differences (Fig. 5h).

**Table 3:** Genetic variation among chickpea-nodulating *Mesorhizobium* strains over geography and altitude

Genetic distance matrices (M)			Mantel/partial statistics for HK		Mantel/partial statistics *nodC*	
M1	M2	Cov.M3	*r*_*(Mantel/partial)*_	*P*	*r*_*(Mantel/partial)*_	*P*
Nucleotides	Geo		0.09327	0.167	− 0.0826	0.586
	Alt		0.3328	0.007**	0.1842	0.144
	SDW		0.08382	0.207		
	Geo	Alt	− 0.01612	0.545	− 0.1732	0.982
Haplotypes	Geo		0.2038	0.009**	− 0.1475	0.847
	Alt		0.05085	0.246	0.2053	0.053.
	SDW		0.03254	0.362		
	Geo	Alt	0.1983	0.009**	− 0.2557	0.299

M is distance matrix, Cov. M is covariate distance matrix, Geo is geographic distance, Alt is altitude, SDW is shoot dry weight, HK is housekeeping genes. Significance: '***' for *p* < 0.001, '**' for *p* < 0.01, '*' for *p* < 0.05, '.' for *p* < 0.1

**Fig. 6:**
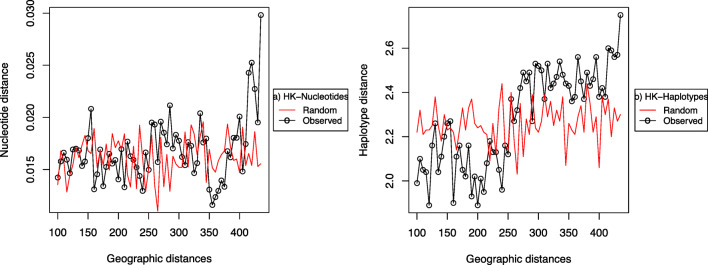
Hypothetical resampling of rhizobia in a sliding window of 400 km. Genetic distances for a pair of strains was estimated at every 10-km distance of sampling and plotted against geographic distance. (**a**) Nucleotide distances and (**b**) locus/haplotype distance. Genetic distance is plotted on the *y*-axis, and geographic distance is plotted on the *x*-axis

Computerized resampling of the rhizobia in a sliding window also confirmed this that no spatial variations occur at higher genomic hierarchy, but the occurrence of non-random variation at haplotypes entail spatial variability at the lower (haplotype) level (Fig. 6). The spatial variability was not considered for the symbiotic genes since they appeared in a single cluster (Fig. 3a,b), recapitulating the dominance of a single symbiotic gene among the test strains. The single symbiovar cluster of the symbiotic genes precluded further analysis.

### Symbiotic Performance and Its Relation to Genetic Diversity

Here, symbiotic performance is defined as the amount of plant dry matter accumulated as a result of association with rhizobia [53] and symbiotic response as the first principal component representing a set of nodulation and biomass traits expected to respond to symbiosis (see Methods). We found that there was a significant variation among individuals for symbiotic response, but not for symbiotic performance (Table S3). This meant that, although symbiotic performance correlated positively with nodule number and weight (*r* = 0.5 and 0.6, respectively), differences in response were mainly driven by differences in nodulation traits rather than shoot biomass. In fact, symbiotic performance was poor overall, with only strain ACRS20 having significantly higher shoot dry weight than the negative control (Dunnett’s test, *p* < 0.05) and performance of ACRS4b and ACRS7b being marginally significant (*p* < 0.06). The majority of strains tested could thus be not shown as effective, despite all of them developing nitrogen-fixing nodules (Table S4). Reanalysis of phenotypic means published by Tena et al. [30] showed significantly reduced shoot dry weight for genospecies II compared to genospecies I (two-sided *p* value = 0.04). A Wilcoxon rank sum test for the same contrast confirmed that overall symbiotic performance was indeed smaller for genospecies II (one-sided *p* value = 0.04), associated with a 16% reduction in biomass. It should be noted however that variation for symbiotic performance in genospecies I is considerable, with a number of strains having very similar performance to those in genospecies II. Not surprisingly therefore, no significant relation was found with either genetic principal coordinates or geography (altitude and geographic principal coordinates).

## Discussion

We investigated genetic diversity, biogeography and possible relationships between *Mesorhizobium* genospecies clusters and symbiotic effectiveness (SE) of 30 strains (Table S1) from Ethiopia, a secondary centre of diversity for chickpea. Chickpea has been reported to be a restrictive host based on cross-inoculation studies [54]. Most studies confirm that it is almost exclusively nodulated by species from the genus *Mesorhizobium* [15, 23, 30, 55] with only few reports including strains from the genus *Sinorhizobium* such as *S. medicae* and *S. meliloti* [56, 57]. Most studies report considerable diversity within *Mesorhizobium* however, with commonly three to four genospecies detected, including *M. ciceri* and *M. mediterraneum*, *M. amorphae*, *M. haukuii*, *M. tianshanense*, *M. muleiense*, *M. opportunistum and M. ﻿wenxiniae* [12, 14, 16, 58–62].

Given Ethiopia’s long history of chickpea cultivation and its status of secondary centre of diversification [23], a large diversity of *Mesorhizobium* species could be expected. Indeed, a recent metagenomic characterization of mesorhizobia from a worldwide samples of chickpea root nodules revealed as many as eight major genetic clades within Central Ethiopia alone [23]. Interestingly, our current sample contains only four of these clades, represented by four genospecies and forming only a single symbiovar. Three of the genospecies, including the dominant one, *M*. *plurifarium*, were assigned to reference species that were originally obtained from tree legumes [45, 63, 64] with only the smallest genospecies assigned to the typical chickpea-nodulating species *M. ciceri* and *M. loti* [14, 16, 59]. The *M. plurifarium* strains isolated from Senegal have relatively a wide host range that ﻿includes *Acacia senegal*, *A. tortilis* subsp. *raddiana*, *A. nilotica*, *A. seyal*, *Leucaena leucocephala*, *﻿Prosopis juliflora* and *Neptunia oleracea* [46, 64, 65], when compared with the chickpea-nodulating strains *M. ciceri* and *M. mediterraneum*. The geographic distribution of the *M. plurifarium* strains were reported to be affected by the annual rainfall patterns, but not the host range [46]. The *M. plurifarium* strain was identified as heat and salt tolerant [46] and probably became the chickpea symbiont in Ethiopia that grows on residual moisture towards the end of the rainy season. The strains belonging to genospecies *M. plurifarium* (cluster I), *Mesorhizobium* sp. WSM3876 (cluster II) and *M. abyssinicae* (cluster III) could reflect the horizontal gene transfer (HGT) from chickpea natural symbionts (*M. ciceri* and *M. mediterraneum*) to the tree legume symbionts. Because they share identical symbiotic genes, and this identity was supposed to occur due to the HGT [14–16]. The HGT between *Mesorhizobium* strains is very common as reviewed by Andrews et al. [21]. The symbiotic (accessary) genes determine biogeographic patterns, host niche and are prone to HGT, while the housekeeping (core) genes determine evolutionary relationships among rhizobia as they transfer vertically between strains [52, 66].

Our sample differed strongly in both diversity and composition from that of Greenlon et al. [23], probably due to different trap hosts used and molecular characterization tools employed. In our case, a locally released cultivar Nattoli was used to trap rhizobia and housekeeping, and symbiotic genes were used for characterization, whereas Greenlon et al. [23] used wildtype and cultivated chickpea plants and characterized the isolates metagenomically directly from nodules or from pure cultures. Within Greenlon et al.’s [23] Central Ethiopian sample, no significant difference in composition was found between cultivated strains and those recovered from nodule metagenomes (*p* = 0.14), suggesting that the difference between the two studies is not due to the inclusion of metagenomic data. The composition of Ethiopian strains differed significantly from the rest of the sample (Chi-squared test *p* < 0.0001), mainly due to the relative abundance of strains from clades 2, 4 and 8 and rarity of the otherwise common clade 7 in Greenlon et al. [23]. Although the *M*. *plurifarium* genospecies was the most common in Greenlon et al.’s [23] sample, the next three most common clades were not or only poorly represented in our sample. The distribution of clades was also more even, translating into high diversity scores. We found no evident explanation for the salient differences in composition and diversity between the two samples, despite the host plants used to trap mesorhizobia from the soils and method of strain characterization employed. Apart from possible population level differences related to prevailing environmental conditions at the time of sampling, the differences may also reflect biases arising from laboratory procedures used to culture the bacteria. The latter would pose a serious challenge when aiming to obtain representative culture collections and deserves particular attention.

Notwithstanding the limited diversity observed in our sample, and despite the fact that individual soils often harbour different genospecies (also reported by [23]), we observed distinct patterns of phylogeographic structure. Genospecies and strains were not distributed randomly with respect to altitude and geographic location, leading to significant correlations between genetic identity at the nucleotide and locus (haplotype) level and geography. There was also significant isolation by distance with respect to locus-level dissimilarity. The latter was only observed over larger distances, suggesting that there is considerable homogenization at the local scale [23]. These findings agree with earlier work in chickpea such as a study in Portugal that reported non-random distribution with respect to soil pH [14] and Greenlon et al.’s global study that reported significant correlation between geography, environmental factor and community composition and genetic distance [23].

From an applied perspective, the most relevant variation is that in symbiotic effectiveness, expressed as the dry matter and protein production by the legume-rhizobium symbiosis [53]. Symbiotic nitrogen fixation in rhizobia is controlled by accessory genes (*nod*, *nif* and *fix*) that are placed in transmissible genetic elements and transfer between rhizobia horizontally as well as vertically [19, 53]. This could potentially translate into a correspondence between symbiotic effectiveness of the rhizobia (phenotypic responses) and their genetic identity at symbiotic or housekeeping loci, which we did not observe in our analyses. The lack of variation in symbiotic effectiveness and the poor performance of the majority of strains reported in the current analysis prompted us to think however that they could be sporadic symbionts, members of species with infrequent occurrence or species with weak (or no) N_2_ fixation ability [67]. Only a few strains (CP123, CP125 and CP148) were assigned with true symbionts of chickpea rhizobia, while most strains grouped with the tree legume nodulating strains which possibly obtained symbiotic genes through HGT. This would be consistent with them indeed being sporadic symbionts of chickpea. Again, the most effective strain in our evaluation was among these, and although we provide some confirmation for a difference in symbiotic performance between genospecies, we show that the link between taxonomy and phenotype is weak at best. Our results are thereby similar to those reported for sets of Portuguese strains, for which an initial relation between plasmid types and symbiotic performance in a relatively small set of isolates [68] could not be confirmed later when a larger collection was tested [14], suggesting that a search for taxonomic markers of effectiveness may remain elusive.

## Conclusion

We report limited genetic diversity in a collection of cultured chickpea-nodulating strains from Central Ethiopia, representing significantly reduced taxonomic diversity compared with previously published samples from the same region. Despite this limited diversity, significant associations between genetics and both elevation and geography were found, associated with geographically limited distribution of three of the four genospecies in our sample. Such patterns are of obvious relevance for future bioprospecting efforts, emphasizing the importance of sampling over wider geographic areas. The fact that different sampling efforts in the same geographic area can have a deviating taxonomic composition also suggests that factors that potentially differ between studies, such as time or even laboratory procedures, can be of relevance. Finally, our results do not provide strong support for the idea that symbiotic effectiveness can be predicted based on taxonomy or geographic origin, implying that phenotypic evaluation of diverse collections of individual strains remains the only method for discovering potentially superior strains.

## Electronic Supplementary Material

ESM 1 (PDF 180 kb)
