# Supplementary material for: Phylogeography and Symbiotic Effectiveness of Rhizobia Nodulating Chickpea (Cicer arietinum L.) in Ethiopia
Source: Microb Ecol. 2020 Oct 24;81(3):703–16. doi: 10.1007/s00248-020-01620-8 (PMC7982387; doi:10.1007/s00248-020-01620-8)
Supplement: Supplementary file 1 — (PDF 180 kb) [file 248_2020_1620_MOESM1_ESM.pdf]

Supplemental materials

Table S1: Passport information and cluster groups of the test strains

Strains	Sites	Lat	Long	Alt	HK Clusters	Sym clusters	SE Clusters	Seq source	SE source	Author
ACRS20	Goro	6.9903	40.4703	1801	I	I	III	Current	Current	Gunnabo
ACRS20a	Goro	6.9903	40.4703	1801	I	I	II	Current	Current	Gunnabo
ACRS20b	Goro	6.9903	40.4703	1801	I	I	NA	Current	NA	Gunnabo
ACRS4	Mojo	8.4258	39.1433	1831	I	I	II	Current	Current	Gunnabo
ACRS4b	Mojo	8.4258	39.1433	1831	I	I	III	Current	Current	Gunnabo
ACRS7	Ada'a	9.5361	38.2931	2529	NA	I	NA	NA	NA	Gunnabo
ACRS7b	Ada'a	9.5361	38.2931	2529	NA	I	NA	NA	NA	Gunnabo
CA1	Taba	7.0000	37.9238	1878	NA	I	NA	NA	NA	Gunnabo
CA10	Taba	7.0000	37.9238	1878	I	I	I	Current	Current	Gunnabo
CP100	Dukem	8.7700	38.8900	1916	I	I	I	Current	Current	Gunnabo
CP102	Dukem	8.7700	38.8900	1916	NA	I	NA	NA	NA	Gunnabo
CP107	Ataye	10.3522	39.9422	1500	I	I	I	Current	Current	Gunnabo
CP129	Ataye	10.3522	39.9422	1500	I	I	II	Current	Current	Gunnabo
CP131	Ataye	10.3522	39.9422	1500	NA	I	NA	NA	NA	Gunnabo
CP141	Legeberi	9.0000	38.9944	2519	NA	I	NA	NA	NA	Gunnabo
CP141a	Legeberi	9.0000	38.9944	2519	I	I	NA	Current	Current	Gunnabo
CP141b	Legeberi	9.0000	38.9944	2519	I	I	NA	Current	Current	Gunnabo
CP141c	Legeberi	9.0000	38.9944	2519	I	I	NA	Current	Current	Gunnabo
CP5	Ele	8.0000	38.4247	1969	NA	I	NA	NA	NA	Gunnabo
CP5b	Ele	8.0000	38.4247	1969	NA	I	NA	NA	NA	Gunnabo
CP63	Jole	8.0000	38.3869	2102	I	I	II	Current	Current	Gunnabo
CP7	Ele	8.0000	38.4247	1969	NA	I	NA	NA	NA	Gunnabo
CP76	Jole	8.0000	38.3869	2102	I	I	II	Current	Current	Gunnabo
CP78	Jole	8.0000	38.3869	2102	I	I	II	Current	Current	Gunnabo
CP88	Jole	8.0000	38.3869	2102	I	I	NA	NA	Current	Gunnabo

CP96	Jole	8.0000	38.3869	2102	I	I	NA	NA	Current	Gunnabo
CPD17	Dalocha	7.8508	38.2273	1961	NA	I	NA	Current	Current	Gunnabo
CPD12	Dalocha	7.8483	38.2307	1964	NA	I	NA	Current	Current	Gunnabo
CPD13	Dalocha	7.8483	38.2307	1964	I	I	NA	Current	Current	Gunnabo
CPD14	Dalocha	7.8483	38.2307	1964	NA	I	NA	Current	Current	Gunnabo
CPD19	Dalocha	7.8511	38.2270	1961	NA	I	NA	Current	Current	Gunnabo
CPJ1	Taba	7.0000	37.9238	1878	I	I	II	Current	Current	Gunnabo
CPJ2	Taba	7.0000	37.9238	1878	NA	I	NA	NA	Current	Gunnabo
CP10	Ele	8.0000	38.4247	1969	II	NA	I	(Tena et al. 2017)	(Tena et al. 2017)	Tena
CP105	Dukem	8.7700	38.8900	1916	I	NA	III	(Tena et al. 2017)	Current	Tena
CP12	Gogeti	8.2298	38.4776	1969	II	NA	I	(Tena et al. 2017)	(Tena et al. 2017)	Tena
CP123	Seladingay	9.9457	39.6153	2901	IV	NA	II	(Tena et al. 2017)	(Tena et al. 2017)	Tena
CP125	Seladingay	9.9457	39.6153	2901	IV	NA	II	(Tena et al. 2017)	(Tena et al. 2017)	Tena
CP130	Ataye	10.352	39.9422	1500	I	I	III	(Tena et al. 2017)	Current	Tena
CP132	Ataye	10.352	39.9422	1500	NA	NA	NA	NA	Current	Tena
CP134	Asfachew	9.9107	39.8100	1878	I	NA	III	(Tena et al. 2017)	Current	Tena
CP136	Armania	9.8498	39.7872	2374	I	NA	III	(Tena et al. 2017)	Current	Tena
CP148	Legeberi	9.0000	38.9944	2519	IV	NA	II	(Tena et al. 2017)	(Tena et al. 2017)	Tena
CP151	Aleltu	9.1953	39.1453	2639	I	NA	III	(Tena et al. 2017)	(Tena et al. 2017)	Tena
CP30	Taba	7.0000	37.9238	1878	II	NA	I	(Tena et al. 2017)	(Tena et al. 2017)	Tena
CP41	Taba	7.0000	37.9238	1878	III	NA	III	(Tena et al. 2017)	(Tena et al. 2017)	Tena
CP49	Taba	7.0000	37.9238	1878	III	NA	III	(Tena et al. 2017)	(Tena et al. 2017)	Tena
CP51	Bodity	6.9309	37.8415	2096	II	NA	I	(Tena et al. 2017)	(Tena et al. 2017)	Tena
CP69	Ele	8.0000	38.4247	1969	II	NA	I	(Tena et al. 2017)	Current	Tena
CP8	Ele	8.0000	38.4247	1969	II	NA	III	(Tena et al. 2017)	(Tena et al. 2017)	Tena
CP84	Akaki	9.0000	38.6975	2416	I	NA	I	(Tena et al. 2017)	(Tena et al. 2017)	Tena
CP98	Akaki	9.0000	38.6975	2416	I	NA	II	(Tena et al. 2017)	Current	Tena
WSM1271	NA	NA	NA	NA	IV	NA	NA	NA	NA	Greenlon
ORS3356	NA	NA	NA	NA	Ib	NA	NA	NA	NA	Greenlon

ORS3365-1	NA	NA	NA	NA	Ib	NA	NA	NA	NA	Greenlon
STM8773	NA	NA	NA	NA	Ib	NA	NA	NA	NA	Greenlon
isolate_1	NA	NA	NA	NA	U	NA	NA	NA	NA	Greenlon
AA22	NA	NA	NA	NA	U	NA	NA	NA	NA	Greenlon
L2C084A000	NA	NA	NA	NA	U	NA	NA	NA	NA	Greenlon
L48C026A00	NA	NA	NA	NA	U	NA	NA	NA	NA	Greenlon
LCM_4577	NA	NA	NA	NA	Ib	NA	NA	NA	NA	Greenlon
ORS3359	NA	NA	NA	NA	Ib	NA	NA	NA	NA	Greenlon
WSM1284	NA	NA	NA	NA	IV	NA	NA	NA	NA	Greenlon
WSM1293	NA	NA	NA	NA	IV	NA	NA	NA	NA	Greenlon
WSM1497	NA	NA	NA	NA	IV	NA	NA	NA	NA	Greenlon
WSM256161	NA	NA	NA	NA	U	NA	NA	NA	NA	Greenlon
WSM2876	Eritrea	NA	NA	NA	II	NA	NA	NA	NA	Greenlon
WYCCWR_10019_C141	NA	NA	NA	NA	U	NA	NA	NA	NA	Greenlon
SDW018_C743	NA	NA	NA	NA	U	NA	NA	NA	NA	Greenlon
ca181	NA	NA	NA	NA	U	NA	NA	NA	NA	Greenlon
CC1192	NA	NA	NA	NA	IV	NA	NA	NA	NA	Greenlon
CMG6	NA	NA	NA	NA	IV	NA	NA	NA	NA	Greenlon
WSM4083	NA	NA	NA	NA	IV	NA	NA	NA	NA	Greenlon
USDA_3392_C520	NA	NA	NA	NA	U	NA	NA	NA	NA	Greenlon
CGMCC_1.11022T	NA	NA	NA	NA	U	NA	NA	NA	NA	Greenlon
ORS1032T	NA	NA	NA	NA	Ib	NA	NA	NA	NA	Greenlon
WSM1271	NA	NA	NA	NA	IV	NA	NA	NA	NA	Greenlon

Where: Lat is latitude, Long is longitude, Alt is altitude, HK is housekeeping genes sequences, Sym stands for symbiotic genes *nifH* and *nodC*, SE is symbiotic effectiveness and Seq is sequences. Roman numbers I, II, III, IV represent cluster numbers 1, 2, 3, 4; Current means strains that were sequenced or evaluated for symbiotic effectiveness by this study

Table S1: Effects of altitude and geographic components on nucleotide and haplotype variation

Response	Factors	Mean Sq	DenDF	F value	Pr(>F)
Nucleotides					
pc1	Alt	0.00093	9.6716	10.5659	0.0091 **
	geo.pc1	0.00009	9.382	0.9824	0.3465
	geo.pc2	0.00001	7.8146	0.2223	0.6502
pc2	Alt	0.00001	11.2248	0.0985	0.7595
	geo.pc1	0.00033	10.5361	5.606	0.0382 *
	geo.pc2	0.00009	8.9903	1.4608	0.2576
pc3	Alt	4.44E-06	16.239	0.3726	0.5500
	geo.pc1	1.34E-05	14.143	1.1231	0.3070
	geo.pc2	2.06E-07	13.199	0.0173	0.8974
Locus (haplotypes					
pc1	Alt	0.11872	8.5433	0.1333	0.7239
	geo.pc1	2.87120	7.861	3.2247	0.1109
	geo.pc2	0.00755	6.6524	0.0085	0.9293
pc2	Alt	0.00001	13.66	0	0.9956
	geo.pc1	0.22777	12.597	0.5136	0.4866
	geo.pc2	0.26903	11.05	0.6067	0.4524
pc3	Alt	2.56329	13.747	9.1706	0.0092 **
	geo.pc1	0.14523	11.736	0.5196	0.4851
	geo.pc2	0.39045	10.897	1.3969	0.2623
pc4	Alt	0.20913	35	1.04	0.3148
	geo.pc1	0.05445	35	0.2708	0.6061
	geo.pc2	2.05496	35	10.2194	0.0029 **

Where: pc is principal components, Alt is altitude, geo is geographic distance

Table S2: Effects of altitude and geographic components on nucleotide and haplotype variation of reduced *Mesorhizobium* strains

Response		Sum Sq	Mean Sq	DenDF	F value	Pr(>F)
Nucelo.pc1	Alt	0.00003774	0.00003774	11.2488	0.5763	0.46337
	geo.pc1	0.00041683	0.00041683	10.0292	6.3653	0.03017*
	geo.pc2	0.00011197	0.00011197	8.4086	1.7099	0.22562
Nucelo.pc2	Alt	1.62E-06	1.62E-06	15.32	0.0967	0.76
	geo.pc1	1.02E-05	1.02E-05	12.862	0.6099	0.4489
	geo.pc2	3.59E-07	3.59E-07	11.803	0.0215	0.8859
Nucelo.pc3	Alt	2.69E-06	2.69E-06	32	6.7987	0.01374*
	geo.pc1	1.18E-06	1.18E-06	32	2.9743	0.09424.
	geo.pc2	4.48E-09	4.48E-09	32	0.0113	0.9159
haplo.pc1	Alt	2.3142	2.3142	10.2805	2.6653	0.13277
	geo.pc1	7.7908	7.7908	7.7795	8.9729	0.01775*
	geo.pc2	0.3568	0.3568	7.3659	0.4109	0.54094
haplo.pc2	Alt	0.54398	0.54398	14.637	1.0966	0.312
	geo.pc1	0.00868	0.00868	12.667	0.0175	0.8968
	geo.pc2	0.08167	0.08167	11.247	0.1646	0.6925
haplo.pc3	Alt	0.03626	0.03626	16.659	0.111	0.7431
	geo.pc1	0.4935	0.4935	12.954	1.5113	0.2408
	geo.pc2	0.17235	0.17235	13.008	0.5278	0.4804

Table S3: Effects of rhizobia inoculation on chickpea plant nodulation, dry matter accumulation and symbiotic response

Response	Factors	Df	Sum Sq	Mean Sq	F value	Pr(>F)
NN	Rep	2	3788.8	1894.39	4.1444	0.0245079 *
	Strain	17	28359.3	1668.2	3.6496	0.0006425 ***
	Residuals	34	15541.2	457.09		
NDW	Rep	2	14781	7390.3	3.0299	0.06153 .
	Strain	17	234653	13803.1	5.659	9.101e-06 ***
	Residuals	34	82931	2439.1		
RDW	Rep	2	3482	1741	0.198	0.8213
	Strain	17	216268	12721.6	1.4471	0.1753
	Residuals	34	298899	8791.2		
SDW	Rep	2	62480	31240	2.3471	0.111
	Strain	17	297185	17482	1.3134	0.2426
	Residuals	34	452554	13310		
SE	Rep	2	1836.2	918.08	2.3471	0.111
	Strain	17	8733.6	513.74	1.3134	0.2426
	Residuals	34	13299.5	391.16		
SymRes	Rep	2	10.343	5.1713	4.1856	0.0237101 *
	Strain	17	78.374	4.6102	3.7315	0.0005303 ***
	Residuals	34	42.007	1.2355		

Where NN = nodule number; NDW = Nodule dry weight; RDW = root dry weight; SDW = shoot dry weight; SE = symbiotic performance or effectiveness; SymRes = symbiotic response (measured as a principal component 1 of responses NN, NDW, RDW and SDW).

Table S4: Mean separation of chickpea shoot dry weight, nodulation and symbiotic effectiveness (SE) using LSD

	SDW	NN	NDW	RDW	SE%	SE rate
N+	0.987^{a}	0.00^{c}	0.000^{e}	0.720^{a}	100.00^{a}	N-fertilised
ACRS20	0.660^{ab}	64.67^{abc}	0.167^{abc}	0.390^{bc}	44.00^{ab}	e
ACRS4b	0.643^{ab}	81.67^{a}	0.193^{ab}	0.380^{bc}	41.14^{ab}	e
CP130	0.617^{b}	56.67^{abc}	0.133^{abcde}	0.437^{abc}	36.57^{b}	e
ACRS20a	0.603^{b}	50.67^{abc}	0.167^{abc}	0.447^{abc}	34.29^{b}	e
CP129	0.570^{b}	63.00^{abc}	0.113^{abcde}	0.387^{bc}	28.57^{b}	e-
ACRS4	0.567^{b}	77.33^{a}	0.240^{a}	0.447^{abc}	28.00^{b}	e-
CP76	0.537^{b}	31.33^{abc}	0.050^{bcde}	0.400^{bc}	22.86$_{b}$	e-
CPJ1	0.537^{b}	22.00^{abc}	0.050^{bcde}	0.390^{bc}	22.86^{b}	e-
CP78	0.530^{b}	49.33^{abc}	0.090^{abcde}	0.473^{ab}	21.71^{b}	e-
CP63	0.527^{b}	68.00^{ab}	0.157^{abcd}	0.400^{bc}	21.14^{b}	e-
CP69	0.493^{b}	25.00a^{bc}	0.030^{cde}	0.420^{bc}	15.43^{b}	e-
CP136	0.467^{b}	24.67^{abc}	0.047^{bcde}	0.410^{bc}	10.86^{b}	e-
CP98	0.467^{b}	58.67^{abc}	0.110^{abcde}	0.397^{bc}	10.86^{b}	e-
CP107	0.463^{b}	37.33^{abc}	0.060^{bcde}	0.410^{bc}	10.29^{b}	e-
CP151	0.440^{b}	10.00^{bc}	0.007^{de}	0.463^{abc}	6.29^{b}	e-
CP100	0.433^{b}	29.33^{abc}	0.050^{bcde}	0.430^{abc}	5.14^{b}	e-
CA10	0.428^{b}	15.33^{abc}	0.056^{bcde}	0.174^{c}	4.23^{b}	e-
CP12	0.417^{b}	5.00^{bc}	0.004^{e}	0.467^{abc}	2.29^{b}	e-
N-	0.403^{b}	0.00^{c}	0.000^{e}	0.393^{bc}	5.71e-06^{b}	Control
DF	38	38	38	38	38	
t	2.024	2.02	2.024	2.024	2.02	
LSD$_{0.05}$	0.181	33.83	0.078	0.149	31.08	

Where SDW (mg) = shoot dry weight measured in milligrams; NN = nodule number; NDW (mg) = Nodule dry weight; N+ = Positive control; N- = negative control; SE = symbiotic effectiveness; LSD = list significant difference; *e* is moderately effective with SE rate between 33%<SE<67% while e- is poor symbiotic effectiveness with SE rate ≤ 33%.
